# Designing cost-efficient randomized trials by using flexible recruitment strategies

**DOI:** 10.1186/1471-2288-12-106

**Published:** 2012-07-24

**Authors:** Menggang Yu, Jingwei Wu, Debra S Burns, Janet S Carpenter

**Affiliations:** 1 Department of Biostatistics & Medical Informatics, School of Medicine and Public Health, University of Wisconsin, Madison, USA; 2 Department of Biostatistics, School of Medicine, Indiana University, 901 West New York Street, Indianapolis, IN 46202, USA; 3 Department of Music and Arts Technology, School of Engineering and Technology, Purdue University, Indianapolis, USA; 4 Department of Adult Health, School of Nursing, Indiana University, 901 West New York Street, Indianapolis, IN 46202, USA

**Keywords:** Interaction, Clinical trial, Cost saving, Recruitment, Power, Sample size

## Abstract

**Background:**

Sample size planning for clinical trials is usually based on detecting a target effect size of an intervention or treatment. Explicit incorporation of costs into such planning is considered in this article in the situation where effects of an intervention or treatment may depend on (interact with) baseline severity of the targeted symptom or disease. Because much larger sample sizes are usually required to establish such an interaction effect, investigators frequently conduct studies to establish a marginal effect of the intervention for individuals with a certain level of baseline severity.

**Methods:**

We conduct a rigorous investigation on how to determine optimum baseline symptom or disease severity inclusion criteria so that the most cost-efficient design can be used. By using a regression model with an interaction term of treatment by symptom severity, power functions were derived for various levels of baseline symptom severity. Computer algorithms and mathematical optimization were used to determine the most cost-efficient research designs assuming either single- or dual-stage screening procedures.

**Results:**

In the scenarios we considered, impressive cost savings can be achieved by informed selection of baseline symptom severity via the inclusion criteria. Further cost-savings can be achieved if a two stage screening procedure is used and there are some known, relatively inexpensively collected, pre-screening information. The amount of total cost savings are shown to depend on the ratio of the screening and intervention costs. In our investigation, we assumed that: 1) the cost of approaching available subjects for screening is constant, and 2) all variables are normally distributed. There is a need to carry out further investigations with more relaxed assumptions (e.g., skewed data distribution).

**Conclusions:**

As cost becomes a more and more prominent issue in modern clinical trials, cost-saving strategies will become more and more important. Strategies, such as the ones we propose here, can help to minimize costs while maximizing knowledge generation.

## Background

Modern research is costly and faces the ever increasing pressures of insufficient funding, yet pressing timelines [1]. Investigators are challenged to maximize the integrity of their studies in the face of many resource restrictions such as limited sample sizes, budgetary constraints, less than ideal follow-up, and short supply of biological samples. These restrictions are often not under the control of the investigators. As a result, studies can be under-powered and end up with inconclusive or inaccurate results. It is imperative for investigators to explicitly consider and save study costs while optimizing the chance of generating valid results and conclusions.

In this article, we consider designing randomized clinical trials where the effect of the targeted treatment or intervention can depend on the baseline symptom or disease severity of enrolled subjects. For example, the treatment may be more effective for subjects with more severe baseline symptoms or disease. In statistical terminology, the treatment interacts with the baseline covariate of severity [2-4].

While the ultimate goal is to establish and quantify such interactions, it can be hard to do so. The main reason is that detecting interaction effects with adequate power usually requires much larger sample sizes than detecting main effects [3,4], consequently making the study infeasible or unduly time consuming in the face of resource restrictions. An alternative and common approach is to base power analysis on detecting marginal or main treatment effects on subjects that meet pre-determined levels of symptom severity (e.g., all subjects have severe symptoms). This ensures that a manageable sample size can be used to conduct the study in a timely manner. Model based approaches for interaction are usually specified as a secondary analysis. For example, instead of conducting a study to demonstrate the interaction effect of an intervention on reducing various levels of symptom severity, a smaller study may be possible due to larger marginal effects of the intervention in a more severe subpopulation. The establishment of such an effect is scientifically meaningful but less useful to clinicians who need to administer treatments to individuals with varying levels of symptom severity.

On the other hand, it can be more difficult and costly to recruit subjects due to the stricter eligibility criteria for severity because it implies more screening. If screening costs are substantial, the total cost of the study may not be reduced. Also setting too high of a baseline severity requirement can increase screening costs because of the added difficulty of identifying a restricted pool of eligible subjects (e.g., lower percentage of subjects with severe symptoms). Thus a trade-off must be made so that resources can be allocated optimally between screening and treating subjects. However in most studies, the determination for the cut-off point for inclusion criteria seems to be based on convenience or intuition, not on mathematical rigor.

To put the cost consideration concretely, consider a recent clinical study [5]. The study needs to cover the salary of recruiter, screenor, intervenor, data collector, medical record abstractor; it also needs to cover the cost of enrollment or patient consent, data monitoring and data entry, drugs or supplies, travel to field - mileage for follow up and many more. A detailed cost analysis from [5] reveals that the cost of recruitment for each subject is 28.7% of the total cost for each subject.

One factor influencing screening costs is whether the recruitment process is done in one or two stages. In a two-stage procedure, basic information from potentially eligible subjects is collected via phone calls or mailings from the first stage. Subjects are usually asked about their interest in participation. From the second stage, only subjects who show interests and appear to be good candidates are approached for actual onsite screening or more intensive screening. One-stage procedure either combines these two stages or omits the first stage effort. It may be argued that most recruitment processes are two-stage processes. Separation of the two stages of the recruitment process allows us to further fine tune the cost spending in clinical trials to reduce costs.

The two-stage procedure has been investigated [6], where the authors considered only the cost allocation between the two stages in the recruitment process for financial savings. Our investigation connects recruitment costs with intervention costs. We develop methods to balance these costs to minimize the total costs of clinical trials. In addition, we illustrate our methods using an example comparing costs from various study designs using different inclusion criteria for baseline symptom severity. We also verify our numerical calculation using simulation studies.

## Methods

Throughout the article, we use intervention and treatment interchangeably to mean the same thing. Consider a trial comparing control with intervention. Let *X* be the baseline symptom severity and *Y* be the symptom severity after intervention. Assume the following model for the effect of intervention:

(1) $$Y=\beta_{0}+\beta_{1\;}Trt+\beta_{2\;}X+\beta_{3\;}Trt*X+\varepsilon \text{.}$$

Here, *Trt* is the indicator of randomized intervention which is independent of *X* and ε ~ N(0, σ²). Therefore both *X* and *Y* are random variables. As usual, we assume that ε is independent of X. When *β*_3_ ≠ 0, we say that there is intervention and covariate interaction. Now suppose we enroll patients only if their baseline symptom severity exceeds a threshold *a*. In the final analysis to declare the marginal effect of the intervention, we use a two-sample *t*-test. That is, we fit a marginal model relating *Y* to the intervention:

(2) $$Y=\lambda_{0}+\lambda_{1}\;Trt+e$$

The significance of the intervention effect is based on testing whether λ_1_ = 0. Although the *t*-test still takes the same form algebraically, the statistical distribution needs to consider the fact that we only enroll subjects with *X ≥ a*.

From (2), we have *λ*_*0*_*+ λ*_*1*_*Trt = E[Y|Trt, X ≥ a]*. From (1), *E[Y|Trt, X ≥ a] = β*_*0*_*+ β*_*1*_*Trt + β*_*2*_*E[X|X ≥ a] + β*_*3*_*E[X|X ≥ a]Trt*, By comparing the coefficients of *Trt*, we obtain

(3) $$l_{1}={\text{β}}_{1}+{\text{β}}_{3}E[X|X\geq a]Z$$

This implies when *β*_*3*_ and *β*_*1*_ have the same signs, the magnitude of *λ*_*1*_ is a monotonic function of *a*. Hence a larger effect can be expected from more severe subgroups. Note that if there is no intervention and baseline symptom severity interaction, that is, *β*_*3*_ = 0, then *λ*_*1*_*= β*_*1*_ so the treatment effect can be estimated in unbiased fashion from the reduced marginal model (2).

For simplicity, we consider equal size randomization between the control and intervention groups. Let *n* be the sample size for each group and *power (n, a)* the corresponding power for testing H_0_: λ_1_ = 0 based on enrolled subjects (i.e. those with *X ≥ a*). Then typically we require *power (n, a)* ≥ 90%. For this enrollment sample size, the corresponding screening size for each group is *N*, which is theoretically equal to *n/P(X ≥ a)*. If we break the recruitment into two stages where we also collect information from a pre-screening variable *Z* on top of *X*, we will have corresponding pre-screening size of *M* and screening size of *N* for each group. The relationship among *M*, *N*, and *n* takes a different form. In the two stage procedure, the first stage recruits subjects with *Z ≥ b* to the second stage to obtain X*.* Those with *X ≥ a* are then randomized to either intervention or control. Then mathematically, we have N = M*P(Z ≥ b), n = N*P(X ≥ a | Z ≥ b) = M*P(X ≥ a, Z ≥ b).

### Designs with a One-stage screening procedure

Recruitment using a one-stage procedure depends on the screening variable *X* only. We denote the recruitment, intervention, and placebo costs by *C*_*rec*_, *C*_*trt*_, and *C*_*placebo*_ respectively. The total cost is then *C*_rec_*2 N + *C*_trt_*n + C_placebo_*n. Note that 2 N and 2n are used because we have two groups (intervention and control) and N and n are screening and enrolled sizes for each group. Our problem becomes a mathematical optimization problem: find *n* and *a* such that

(4) $$\left\{ \begin{array}{l} \text{Power}(n,a)\geq 90\%\text{,}\;n/N=P(X\geq a)\\ C_{\text{trt}}*n+C_{\text{placebo}}*n+C_{\text{rec}}*2N\;\text{is}\;\text{minimize} \end{array}\right.$$

We now derive a formula for *power (n,a)*. Let *X*_*1*_ and *X*_*2*_ be the baseline symptom severities and let *Y*_*1*_ and *Y*_*2*_ be the symptom severities after treatment in the control and intervention groups respectively.

The *t*-test for testing *H*_0 _*:λ*_1_ = 0 can be expressed as

$$T=\frac{\overset{―}{Y_{2}}-\overset{―}{Y_{1}}}{\sqrt{\text{Var}(\overset{―}{Y_{2}})+\text{Var}(\overset{―}{Y_{1}})}}\text{,}$$

where ${\overset{―}{Y}}_{1}=n^{-1}\sum_{i=1}^{n}Y_{1i}$and ${\overset{―}{Y}}_{2}=n^{-1}\sum_{i=1}^{n}Y_{2i}$From model (1), the expected value *T* can be obtained as

$$E(T)=\sqrt{n}\frac{\beta_{1}+\beta_{3}E[X|X\geq a]}{\sqrt{\text{Var}(Y_{1}|X_{1}\geq a)+\text{Var}(Y_{2}|X_{2}\geq a)}}$$

and the variance as *Var* (*T*) = 1. Because $\text{Var}(Y_{1}|X_{1}\geq a)=\sigma^{2}+\beta_{2}^{2}\text{Var}(X|X\geq a)$ and $\text{Var}(Y_{2}|X_{2}\geq a)=\sigma^{2}+{\left(\beta_{2}+\beta_{3}\right)}^{2}\text{Var}(X|X\geq a)$, the power level based on *T* for testing $H_{0}:\lambda_{1}=0$ vs. *H*_*1*_*: λ*_*1*_*≠ 0* can be expressed using t-distribution. In moderate to large sample sizes, this power can be well approximated by [7]

(5) $$\text{power}(n,a)=\Phi \left(\sqrt{n}\frac{\beta_{1}+\beta_{3}E(X|X\geq a)}{\sqrt{2\sigma^{2}+\{\beta_{2}^{2}+{\left(\beta_{2}+\beta_{3}\right)}^{2}\}\text{Var}(X|X\geq a)}}-1.96\right)$$

where *Φ*( · ) is the cumulative distribution function (CDF) for a standard normal distribution. To evaluate *power (n,a) *for any given *n* and *a*, we need to know the parameter values of *β*_*0*_*, β*_*1*_*, β*_*2*_*, β*_*3*_*, σ,* and the distribution of *X*. The value of *σ* and the distribution of *X* can usually be approximated from historical data or pilot studies. To specify the values of the regression parameters, noticing that under the assumption of no control effect, we may safely assume *β*_*0*_ = 0 and *β*_*2*_ = 1*.* To solicit values of *β*_*1*_ and *β*_*3*_, we need to obtain the targeted reduction when using at least two different threshold values of *a.* This is natural for investigators to consider due to the interaction nature of the intervention. Specifically, we need to know targeted value λ_11_ if we set $X\geq a_{1}$ and λ_12_ if we set $X\geq a_{2}\text{.}$Then from the equations $\begin{array}{l} \lambda_{11}=\beta_{1}+\beta_{3}E(X|X\geq a_{1})\text{ and }\\ \lambda_{12}=\beta_{1}+\beta_{3}E(X|X\geq a_{2}) \end{array}\text{,}$we can obtain targeted values of *β*_1_ and *β*_3_.

The algorithm for determining the solution to Problem (4) then proceeds as follows. First, for any given *a,* we determine a corresponding *n* such that the power constraint is satisfied. Then, we evaluate the total cost at all possible range of *a* and find the optimum value so that the total cost is minimized.

### Designs with a two-stage screening procedure

Quite often, the screening process involves multiple steps such as 1) identification of potentially eligible subjects via an existing database; 2) initial screening via phone or mail using simple measures to exclude those obviously ineligible subjects; and 3) on-site, or phone, or mail screening using full measures of eligibility criteria.

We consider utilizing the primitive information from steps 1 and 2, which we call the ‘pre-screening’ stage. In many cases, the cost associated with pre-screening is relatively inexpensive and information collected at the pre-screen stage can help offset the higher costs associated with more intensive screening in step 3. The rationale is that if we can use the primitive information to predict a more expensive screening outcome that can depend on many eligibility criteria, then we should be able to reduce the number of intensive screenings. As a result, we may perform more pre-screening in exchange for less intensive screening. Costs may be saved because of such a trade-off, especially if pre-screening is inexpensive. In conducting symptom management studies, the most relevant information collected during pre-screening can be self-reported symptom severity. This is typically rated on a Likert-type scale (none, mild, moderate, severe) or numeric rating scale (0 none to 10 extremely severe).

Assume that *Z* is a primitive covariate collected in the pre-screen stage. In other words, *Z* can be viewed as a surrogate variable for *X.* The two stage procedure only recruits subjects with Z≥b for the second stage intensive screening to obtain *X.* Those with X≥a are then randomized to either intervention or control. Assume that we pre-screen *M* subjects, screen *N* subjects and enroll n subjects for each group. Then mathematically, we have N = M*P(Z ≥ b), n = N*P(X ≥ a | Z ≥ b) = M*P(X ≥ a, Z ≥ b). Denote the prescreening, screening, intervention, and placebo costs are denoted by *C*_*pre*_, *C*_*scr*_, *C*_*trt*_ and *C*_*placebo*_ respectively. The total cost is *C*_pre_*2 M + *C*_scr_*2 N + *C*_trt_*n + C_placebo_*n. Hence our problem becomes a mathematical optimization problem: find *n*, *a*, and *b* such that

(6) $$\left\{ \begin{array}{l} \text{Power}(n,a)\geq 90\%\text{,}\;n=N*P(X\geq a|Z\geq b)=M*P(X\geq a,Z\geq b)\\ C_{\text{trt}}*n+C_{\text{placebo}}*n+C_{\text{scr}}*2N+C_{\text{pre}}*2M\;\text{is minimized} \end{array}\right.$$

Note that the total cost can be written as

$$n\left\{C_{\text{trt}}+C_{\text{placebo}}+\frac{2C_{\text{scr}}}{P(X\geq a|Z\geq b)}+\frac{2C_{\text{pre}}}{P(X\geq a,Z\geq b)}\right\}.$$

Because *power (n,a)* does not involve *b,* for any given *n* and *a,* we can optimize the total cost as a function of *b.* Then the algorithm for the solution to (6) can proceed similar to the Problem (4). In evaluating the total cost, we need to know the joint distribution of *(X, Z)*. The joint distribution may be estimated from existing data or via a prior specification based on literature search [8]. In our case, we assume that *(X, Z)* are jointly normal variables. The correlation coefficient between *X* and *Z* is denoted by *ρ*.

## Results

In this section, we first illustrate our methods using an example. We then confirm the accuracy of our numerical calculations in some selected scenarios by comparing our numerical calculations with simulation results. All our computation is based on the R language version 2.14.1 (*http://cran.r-project.org/*).

### A symptom severity example

Hot flashes are frequent, severe, bothersome events that interfere with daily life for millions of breast cancer survivors (BCS) and menopausal women without breast cancer (MW). Hot flashes have been associated with mood disturbance, negative affect, and sleep disturbance in BCS and with sleep disturbance in MW [9,10]. Although hormone therapy is an effective treatment, it is contraindicated for BCS and no longer acceptable to many MW because of shifts in its risk-benefit ratio uncovered by the Women’s Health Initiative study [11]. Unfortunately, the scientific basis for non-hormonal management of hot flashes is limited [12,13].

We are therefore interested in a particular non-hormonal hot flash treatment that has the potential to reduce hot flash symptoms. A randomized clinical trial needs to be designed to compare the intervention with a control condition. One of the primary outcomes of the study is hot flash severity which is measured from 0 (not at all) to 10 (extremely). The effect of this particular intervention is hypothesized to be greater for those with more severe baseline hot flashes. However direct testing of such interaction term would require a very large sample size. As is commonly done, we intend to detect marginal treatment effects on a selected subset of eligible subjects so that a manageable sample size can be used to conduct the study in a timely manner. Yet, determination of such a subset is a common issue in hot flash clinical trials [5,8,13-15]. Although such arbitrariness in determining inclusion criteria has limited impact on the validity of statistical testing of the marginal effect, it can affect the cost and timeline of a resulting trial, depending on the actual costs of recruitment and intervention. Intervention usually involves applying the intervention and following up with enrolled subjects. Recruitment usually involves costs of accessing databases, phone interviewing or mailing, on-site screening, etc.

In our example, we have observed that for BCS, about 47% of total costs were spent on recruitment while for MW, about 17% was spent on recruitment. Because cost saving percentages are of real interest when comparing different designs, for simplicity, we take unit intervention cost *C*_*trt*_*=* $700, unit placebo cost *C*_*placebo*_*=* $700, and unit recruitment cost *C*_*rec*_*=* $300. Thus we assume 30% of total cost is on recruitment. In the two stage screening procedure, we further break down recruitment cost *C*_*rec*_ into two parts: onsite screening cost *C*_*src*_*=* $200 and pre-screening cost *C*_*pre*_*=* $100.

**Figure 1:**
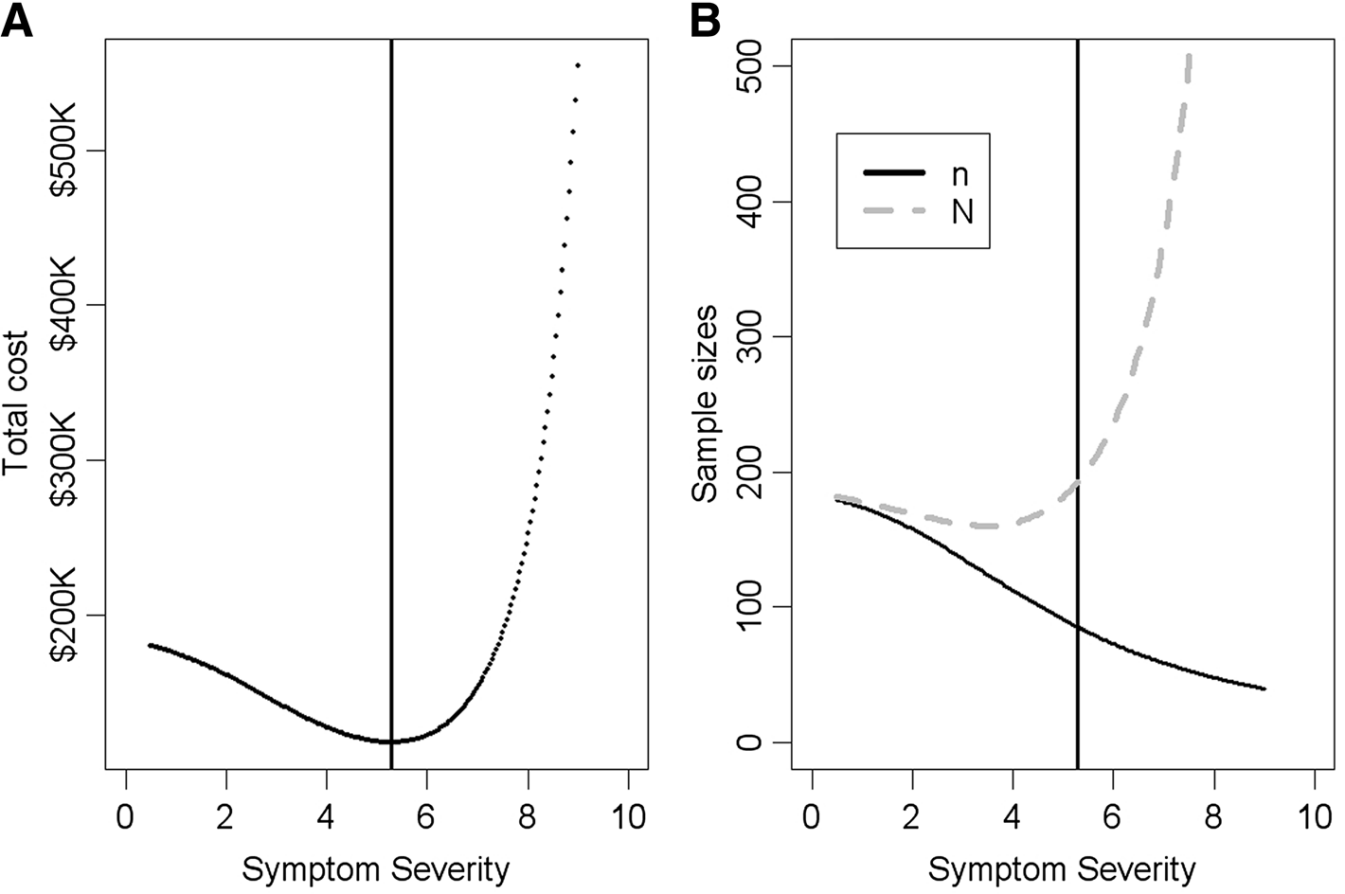
**Plots of total costs (A) and sample size (B) as a function of the symptom severity threshold used for inclusion criteria.** The vertical line indicates the optimum threshold for minimum total cost. The treatment sample size (solid line in B) decreases as the symptom severity threshold increases, whereas the screening size (dashed line in B) is curvilinear as a function of the symptom severity threshold

Using published data, we assume baseline hot flash severity is normally distributed. We take X ~ N(5, 2²). We set the regression coefficients β_0_ = 0, β_1_ = -0.2, β_2_ = 1, and β_3_ = -0.25, based on our preliminary analysis of a recently completed hot flash study. Finally, we take *σ = 2.5* as the standard deviation for the error term. Figure 1 illustrates our approach and the corresponding results for the one stage procedure from Section 3.1. The optimum threshold for our baseline symptom severity inclusion criteria is found to be a = 5.3, close to the mean (see Figure 1A). The treatment sample size *n* (solid line in Figure 1B) decreases as the threshold increases, whereas the screening size *N* (dashed line in Figure 1B) decreases first and then increases. The rounded optimum screening and treatment sizes are 193 and 86 respectively. The optimum cost is $118,100. If the cut-off is taken as 4 (cut off for moderate severity), the rounded optimum screening and treatment sizes are 163 and 113 with the corresponding total cost of $128,000. If the cut-off is taken as 7 (cut off for severe symptoms), the rounded optimum screening and treatment sizes are 370 and 60 with the corresponding total cost of $153,000. Compared with the optimum design, the cost increases are 8.4% and 29.5% respectively.

**Figure 2:**
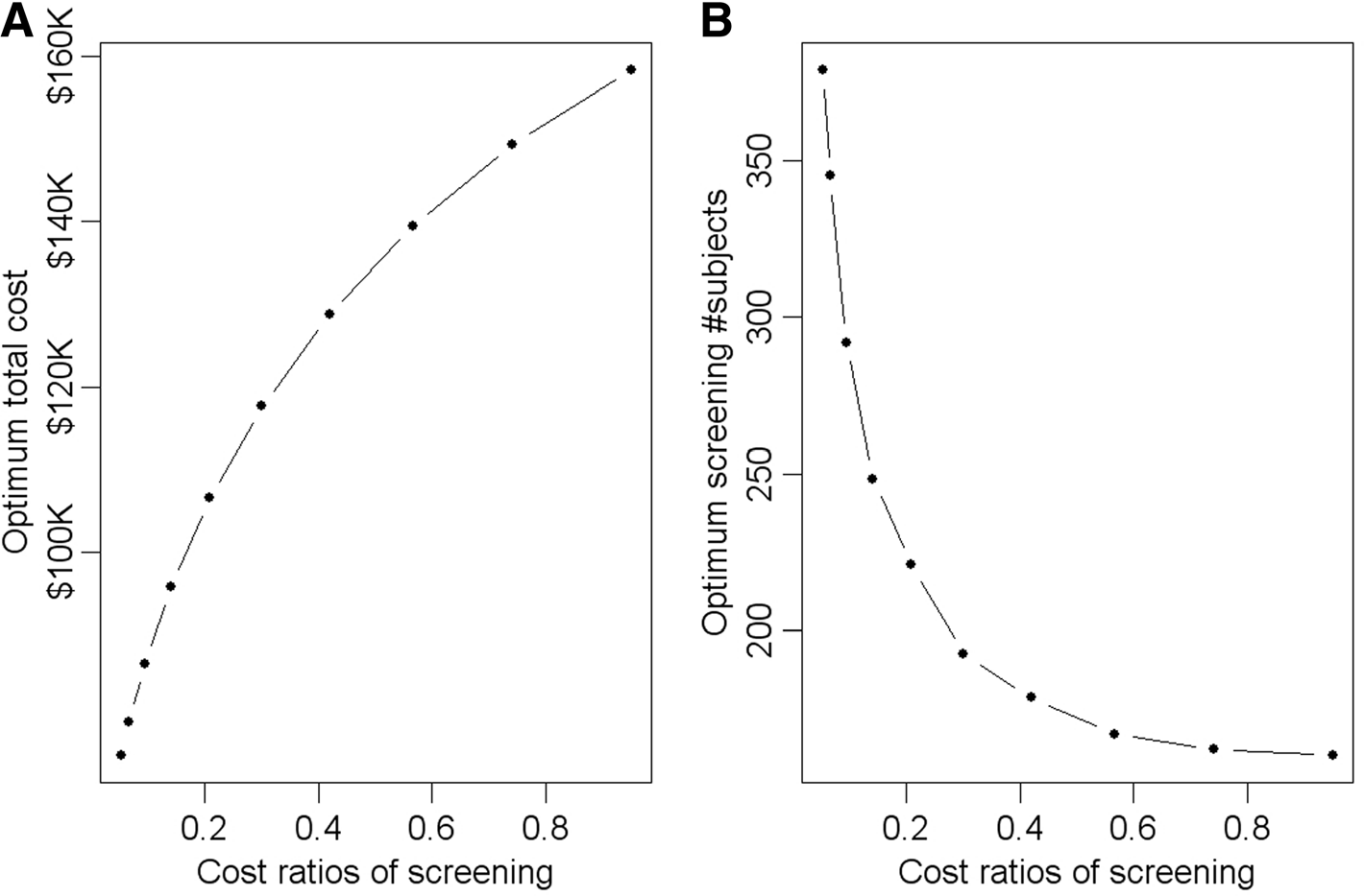
**Plots of optimum total costs (A) and optimum screening sample sizes (B) as a function of cost ratios of screening.** The optimum total costs increase as the cost of screening is weighted more heavily in comparison to the cost of intervention (in A), whereas the screening size decreases (in B)

To assess how the optimum solutions change as a function of varying screening costs, we also change the screening costs while fix the sum of the screening and intervention costs as $1000. As expected, when the screening cost comprises a bigger proportion of the two costs, the optimum total cost increases (Figure 2A). On the other hand, the optimum screening sample size decreases (Figure 2B).

**Figure 3:**
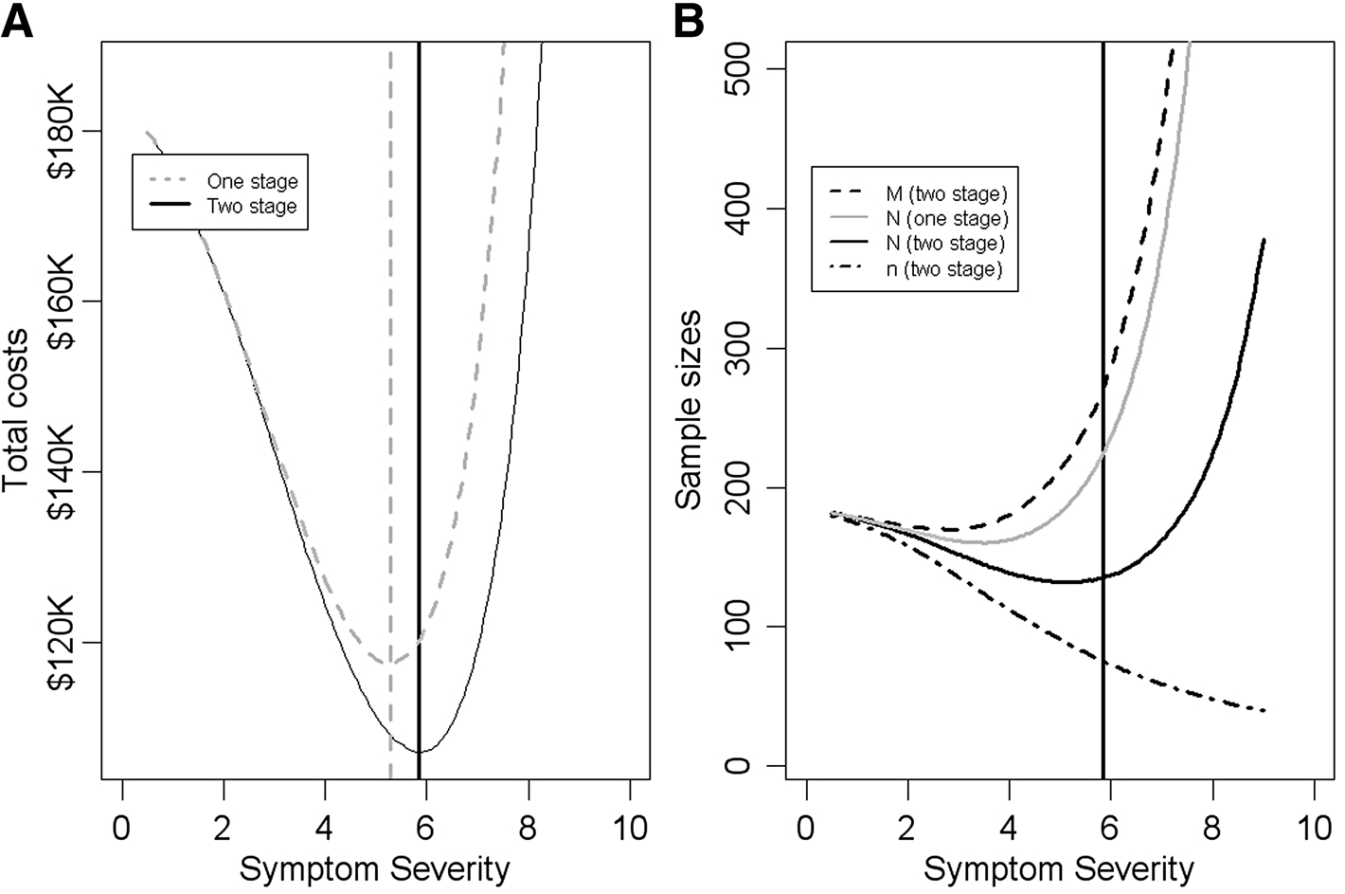
**Plots of optimum total costs (A) and sample sizes (B) as a function of screening severity threshold *a*.** The solid vertical lines indicate the optimum screening severity threshold for minimum total cost under the two stage procedure. The dashed vertical line indicates the optimum screening severity threshold for minimum total cost under the single stage procedure. In B, the gray solid line is the screening sample size under the single stage procedure.

Figure 3 plots optimum total costs (Figure 3A) and various sample sizes (Figure 3B) as a function of screening threshold *a* .The figure compares the two stage and single stage screening procedures. Here we assume the correlation *ρ* between *Z* and *X* is 0.7. The optimum threshold for prescreening and screening cut-offs are *a = 5* and *b = 5*.8 under the two stage screening procedure (Figure 3A). This leads to the rounded-up optimum prescreening, screening and treatment sizes of 272, 136, and 76 respectively, with a minimum total cost of $107,600. Compared with the single stage screening procedure, the cost saving from the two stage screening procedure is $10,500 (or 8.9%).

As expected, the treatment sample size *n* (solid line in Figure 3B) decreases as the threshold increases, whereas the screening size *N* and prescreening size *M* (dashed line in Figure 3B) decrease first and then increase under both procedures. Note that the treatment sample size curve is the same under both procedures as it is completely determined from the power function constraint, *power (n,a) ≥* 90%. We also note that the screening size curve under the single stage procedure lies in-between the prescreening and screening sample size curves under the two stage procedure.

**Figure 4:**
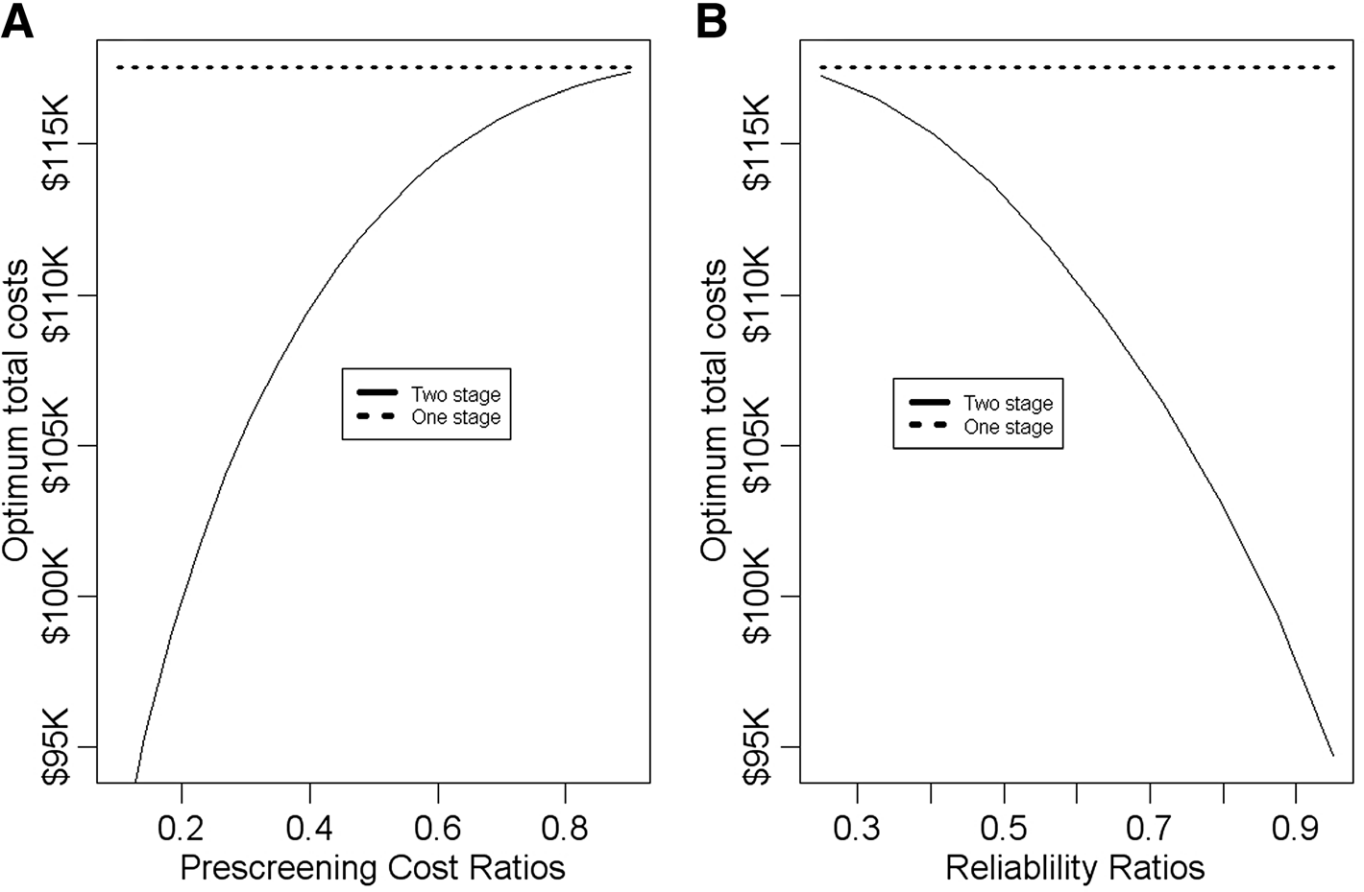
**Plots of optimum total costs as a function of prescreening cost ratios (A) and of the correlation coefficient ρ (B).** The solid lines correspond to the two stage screening procedure and the dashed line to the single stage procedure.

To further investigate how the cost saving change with the cost of prescreening procedure *C*_*pre*_, we vary the ratio while keeping the sum *C*_*pre*_ + *C*_*scr*_ fixed at $300. Figure 4A plots optimum total costs as a function of (*C*_*pre*_ + *C*_*scr*_)^−1^*C*_*pre*_. We see that the smaller the ratio, the more is the cost saving. This is intuitively clear because smaller ratios of the prescreening costs translate to more cost efficient use of the information collected from the prescreening stage.

To investigate how the cost saving change with the quality of surrogacy of *Z*, we vary the correlation coefficient *ρ* while keeping the costs as *C*_*trt*_*=* $700, *C*_*placebo*_*=* $700, *C*_*scr*_*=* $200, and *C*_*pre*_*=* $200. Figure 4B plots optimum total costs as a function of the correlation coefficient *ρ*. We see that the larger the value of the correlation coefficient, the more the cost savings. This is also intuitively clear because the higher value of the correlation coefficient translates to more relevancy in information collected from the prescreening stage.

### Simulation study

In our simulation, we investigate both empirical size and power of the resulting one stage and two stage screening procedures. In particular, we generate X ~ N(5, 2²) and Z ~ N(5, 2²) with different correlation ρ. The regression error standard deviation is taken as σ = 2.5. The test sizes are calculated with the regression coefficients β_0_ = 0, β_1_ = 0, β_2_ = 1, and β_3_ = 0, whereas the powers are calculated with β_0_ = 0, β_1_ = -0.2, β_2_ = 1, and β_3_ = −0.25. These parameters were set to mimic the results obtained from a recent study [5]. The ratio of the prescreening cost over the screening cost also varies. We also compare the costs from numerical calculations and from simulated results. The intervention and placebo costs are fixed at *C*_*trt*_*= C*_*placebo*_*=* $700, and the sum of prescreening and screening costs are fixed at *C*_*scr*_ + *C*_*pre*_ = $300.

Numerical calculations are based on alpha level of 0.05 and power level of 90%. In prescreening, screening, and treatment sample size determination, we use the least integers that are larger than calculated sample sizes (which can be a non-integer number). The prescreening variable *Z*, screening variable *X*, and the actual treatment and outcome are simulated in sequel according to the determined cut-offs. Simulation results are shown in Table 1. The actual power levels and test sizes from simulated data are matching the calculated alpha and power levels. The actual costs from simulated data are slightly higher than the calculated costs due to the fact that we up-round the calculated prescreening, screening, and treatment sample sizes to integer values. The corresponding marginal treatment values λ_1_ are also provided.

**Table 1:** **Simulated results for single stage and two stage screening procedures**^**&**^

		**Calculated costs**		**Simulated costs**		**Simulated actual power**		**Simulated actual type I error**		**Marginal treatment effects (λ**_1_)	
$\frac{C\text{pre}}{C\text{pre}+C\text{scr}}$**†**	***ρ* ‡**	**Single stage**	**Two stage**	**Single stage**	**Two stage**	**Single stage**	**Two stage**	**Single stage**	**Two stage**	**Single stage**	**Two stage**
0.10	0.70	$117.5 K	$90.8 K	$118.3 K	$92.0 K	0.91	0.91	0.05	0.05	0.27	0.33
0.20	0.70	$117.5 K	$99.9 K	$118.4 K	$101.5 K	0.90	0.90	0.06	0.05	0.27	0.31
0.30	0.70	$117.5 K	$105.6 K	$118.3 K	$108.3 K	0.89	0.91	0.05	0.05	0.27	0.30
0.40	0.70	$117.5 K	$109.5 K	$118.3 K	$112.0 K	0.89	0.91	0.06	0.06	0.27	0.30
0.50	0.70	$117.5 K	$112.4 K	$118.4 K	$113.4 K	0.90	0.91	0.04	0.05	0.27	0.29
0.33	0.30	$117.5 K	$116.8 K	$118.5 K	$118.9 K	0.90	0.91	0.05	0.05	0.27	0.28
0.33	0.45	$117.5 K	$114.5 K	$118.5 K	$115.8 K	0.89	0.90	0.04	0.05	0.27	0.28
0.33	0.60	$117.5 K	$110.5 K	$118.5 K	$113.3 K	0.89	0.91	0.05	0.05	0.27	0.30
0.33	0.75	$117.5 K	$105.2 K	$118.3 K	$107.6 K	0.90	0.91	0.05	0.06	0.27	0.30
0.33	0.90	$117.5 K	$98.0 K	$118.5 K	$99.8 K	0.90	0.91	0.06	0.04	0.27	0.31

&“Single stage” screening procedure is defined in Section 2.1 where the screening process is viewed as one inseparable part of the clinical trial, this is in contrast to “two stage” screening procedure where the screening process is broken into a prescreening stage and screening stage (usually intensive or expensive).

†Ratio of prescreening costs to total screening costs. Higher ratio means prescreening costs make up a greater percentage of total screening costs.

‡Correlation coefficient between prescreening and screening variables.

## Conclusions

As cost becomes a more and more prominent issue for conducting modern clinical trials, cost-saving strategies should be taken as a priority measure for successful conduct of trials. Various aspects of trials should be considered in the design stage so that the resulting trials are speedy and economical.

We considered cost-efficient designs for clinical trials in the case when the effect of an intervention may depend on baseline symptom severity. Optimum baseline severity threshold for inclusion criteria can be determined based on an objective cost function so that the study can be designed at lower costs. In practice the mathematically determined threshold may be rounded to a practical value near the optimum solution. This round-up leads to a ‘nearly’ optimum solution and usually has limited impact on costs. We have presented our results using 90% power. When 80% power is used, similar results in terms of relative cost savings were observed.

When the recruitment process consists of two stages: a prescreening stage from either database search or mail/phone contact of potential subjects and an active (usually intensive or expensive) screening stage, we demonstrated that further cost saving can be achieved by utilizing information collected from a pre-screening stage if possible.

The interaction parameters are obtained by asking for targeted improvement or effect of intervention under two cut-off points for baseline measures. When there are concerns about such solicitation, sensitivity analysis can be done to clarify the robustness of our results. When there are different cost-structures associated with the intervention and control groups, our method can be generalized.

Although a *t*-test without using X is used as the main analysis due to the pilot nature of early studies and relative smaller sample size. One should also perform a secondary analysis using X in the model. Such analysis should be performed once the marginal *t*-test is significant.

We also note that our formulation can be easily adapted to different group sizes. Our problem becomes the following mathematical optimization problem: find *n*_*1*_*, n*_*2*_*,* and *a* that satisfy *Power(n*_*1*_*, n*_*2*_*, a) ≥ 90%,* such that *n*_*1*_*/N*_*1*_*= P(X ≥ a), n*_*2*_*/N*_*2*_*= P(X ≥ a)* and *C*_trt_*n_*1*_*+ C*_placebo_*n_*2*_*+ C*_rec_*(N_*1*_*+ N*_*2*_*)* is minimized*.* Here *Power(n*_*1*_*, n*_*2*_*, a)* has explicit expression that can be derived similar to formula (5) on page 9 but using *t*-test with unequal group sizes.

Finally, we list some of limitations in the current investigation. First for simplicity, we only considered normally distributed outcome and screening variables. There are certainly other cases of non-normal continuous variables or discrete variables. Thus, there is a need to carry out a similar investigation in other situations to make the methodology more generalizable. Second, because the proposed study design in this article only recruits subjects with X passing certain threshold, generalizability and interpretation of the conclusion is limited to subjects within same subset. Third, we also assumed that the cost of approaching available subjects for screening is the same throughout this article. Such may not be the case. Costs can change depending on where the subjects are approached. There can also be a limited number of subjects for screening. In such cases, the cost of obtaining extra subjects can be prohibitive. These situations can be dealt with using a variant screening cost function which can depend on the study sites, number of approached subjects, and/or number of sites, etc. When such a function can be specified, the method in this article is directly applicable with slightly more involved computation.

In summary, we demonstrate a methodology for designing clinical trials for normally distributed continuous baseline values of symptom or disease severity. This methodology can be used to determine the optimum sample size while balancing costs.

## Competing interests

The authors declare that they have no competing interests.

## Authors' contributions

MY did most of the computation and contributed to the design of the study; JW, DB, and JC contributed to the design of the study; all authors contributed to the analysis and writing. All authors read and approved the final manuscript.

## Pre-publication history

The pre-publication history for this paper can be accessed here:

http://www.biomedcentral.com/1471-2288/12/106/prepub
